# Inhalation aromatherapy for the treatment of comorbid insomnia: a systematic review and meta-analysis

**DOI:** 10.3389/fpsyt.2025.1485693

**Published:** 2025-03-31

**Authors:** Hu-Bo Chen, Yi-Dan Zhang, Yang Qin, Hong-Yu Jiang, Li-Na Wang, Wei Gu

**Affiliations:** ^1^ College of Basic Medicine, Naval Medical University, Shanghai, China; ^2^ Department of Traditional Chinese Medicine, Naval Medical University, Shanghai, China

**Keywords:** aromatherapy, comorbid insomnia, insomnia, randomized controlled trial, meta-analysis

## Abstract

**Objective:**

Meta-analysis can pool multiple studies to explore a particular area in depth, therefore this method was used to explore the clinical efficacy of inhalation aromatherapy for the treatment of comorbid insomnia and provide an empirical evidence for clinical treatment.

**Methods:**

The PubMed, Web of Science, CNKI Database, Wanfang Database, and VIP Database were searched for randomized controlled trials on aromatherapy for the treatment of comorbid insomnia from inception to August 30, 2023. RevMan 5.3 was used for meta-analysis of the results.

**Results:**

A total of 27 publications involving 2072 patients were included. The results of the meta-analysis showed that inhalation aromatherapy well relieved the symptoms of comorbid insomnia (MD -2.90, 95% CI: -3.85 to -1.95, p<0.00001) and the negative mental state of anxiety (MD -3.97, 95% CI: -5.88 to -2.06, p<0.0001) and depression (MD -9.58, 95% CI: -15.13 to -4.03, p=0.0007) in patients. Included studies were heterogeneous, and the factors influencing the heterogeneity were not identified. These studies commonly presented issues such as lack of blinding, and absence of independent testing for the purity or potency of herbs.

**Conclusion:**

Inhalation aromatherapy can improve the sleep quality of patients with other disease states, with basically no adverse reactions and acceptable safety. Therefore, inhalation aromatherapy is expected to become an indispensable complementary therapy in clinical practice for most diseases. However, high-quality clinical trials are still needed to confirm these findings due to methodological weaknesses in blinding and independent testing for the purity and potency of herbs.

**Systematic review registration:**

https://www.crd.york.ac.uk/prospero/, identifier CRD42023455278.

## 1 Introduction

Sleep quality surveys of different groups of people have shown that insomnia is common and mainly manifests as difficulty in falling asleep or staying asleep, with serious impairment of daytime function (1–5). In clinical practice, the symptoms of insomnia often do not appear alone but are accompanied by other mental or physical disorders. Therefore, insomnia can be divided into primary insomnia and secondary insomnia, but it is difficult to differentiate between the two in clinical practice (6). According to a scientific meeting of the National Institutes of Health, “secondary insomnia” was renamed “comorbid insomnia” (7). Psychological disorders, chronic pain, atrial fibrillation, and HIV infection are risk factors for comorbid insomnia (8–11).

Considering the underlying disease of comorbid insomnia, many drugs for the treatment of insomnia cannot be used in clinical practice (12, 13). There have been many reports that behavioral cognitive therapy, as an emerging field, can stably and effectively improve comorbid insomnia in various diseases, but this method requires much manpower and resources and cannot benefit most patients (14). Therefore, complementary and alternative medicine therapies can have advantages (15). Many young people alleviate their symptoms of comorbid insomnia through complementary and alternative medicine therapy (16).

Inhalation aromatherapy, as a complementary and alternative medicine, involves the absorption of volatile aromatic plant oils into the body through the respiratory mucosa to relieve mental stress, eliminate diseases, and promote human health (17). Studies on the extraction of aromatic oils from natural plants have shown that valerian oil, lavender oil, and chamomile oil can relieve the symptoms of insomnia (18–22). However, studies on insomnia secondary to other diseases have not been collated for systematic evaluation. This study is dedicated to exploring the therapeutic effects of aromatherapy in different disease types, and then determining whether aromatherapy can be applied as a universal clinical adjunct therapy.

To the best of our knowledge, there is no systematic review or meta-analysis summarizing the therapeutic effect of inhalation aromatherapy on comorbid insomnia. Given the high incidence of comorbid insomnia and the limitations of clinical work on it, it is important to accumulate current evidence on the effect of inhalation aromatherapy on comorbid insomnia. Therefore, the purpose of this study was to summarize the efficacy and safety of inhalation aromatherapy in the treatment of comorbid insomnia.

## 2 Materials and methods

### 2.1 Study registration

We conducted and reported this systematic review according to the PRISMA statement (23). This systematic review has been registered (Reg. No. CRD42023455278) in PROSPERO.

### 2.2 Inclusion criteria

#### 2.2.1 Types of studies

All the studies were publicly published randomized controlled trials (RCTs), with language restricted to Chinese or English, on the treatment of comorbid insomnia with inhalation aromatherapy.

#### 2.2.2 Type of interventions

The treatment group was treated with inhalation aromatherapy. Inhalation aromatherapy is defined as a natural therapy in which the volatile oils of aromatic plants are absorbed into the body through breathing to relieve mental stress, eliminate disease, and promote human health. The control group received conventional treatment or placebo treatment.

#### 2.2.3 Type of participants

All subjects met the diagnostic criteria for a disease established by modern medicine, tolerated the volatile oils of aromatic plants used in the study, had no history of using sleeping pills or sedative drugs, had no demand for such drugs during the study, and were in stable condition during the study. There were no other serious complications. All patients were over 18 years old, had a normal sense of smell, and provided informed consent for this study.

#### 2.2.4 Outcome indicators

Primary outcomes: Pittsburgh Sleep Quality Index (PSQI).

Secondary outcomes: (1) Self-Rating Anxiety Scale (SAS); (2) Self-Rating Depression Scale (SDS); (3) PSQI-sleep quality; (4) PSQI-time to falling asleep; (5) PSQI-sleep duration; (6) PSQI-sleep efficiency; (7) PSQI-sleep disorder; (8) PSQI-daytime function.

### 2.3 Exclusion criteria

(1) Nonclinical trial publications such as animal studies and review papers; (2) review papers or meta-analysis papers; (3) repeat publications (the first published was selected); (4) papers that did not clearly state the selection and method of use of aromatherapy; (5) papers with nonstandardized experimental designs or inappropriate use of statistical methods; (6) papers without standards for efficacy evaluation or with unscientific study results.

### 2.4 Literature search strategy

The English databases PubMed and Web of Science and the Chinese databases CNKI Database, Wanfang Database, and VIP Database were searched. The literature search was limited to Chinese and English. The Chinese search terms used were “insomnia (不寐)”, “insomnia (失眠)”, “sleep disorders (睡眠障碍)” “sleep quality (睡眠质量)”, “sachet (香囊)”, “aromatherapy (芳香疗法)”, “plant essential oil (植物精油)”, and “aromatherapy (香薰疗法)”. The English search terms used were “insomnia”, “sleep disorder”, “sleep quality”, “aromatherapy”, and “essential oil”. The retrieval time frame ranged from database inception to August 30, 2023.

### 2.5 Efficacy evaluation criteria

(1) Psychological state evaluation: The subjects selected in this study all had underlying disease. Underlying disease creates a negative psychological state which is closely related to sleep quality. Therefore, it was necessary to evaluate patients’ psychological state. The SAS and SDS were used to evaluate the negative psychology of the patients. (2) Sleep quality: The PSQI was used to evaluate the sleep quality of the patients. A total of 18 items formed seven dimensions, each dimension having 0-3 points, for a total score of 21 points. The total score of the PSQI ranged from 0-21 points.

### 2.6 The GRADE approach

The GRADE (Grading of Recommendations Assessment, Development and Evaluation) approach was applied to evaluate the level of evidence in the included literature (24, 25). The GRADE process includes all important and critical outcomes explicitly. The main domains used to assess the certainty of the evidence are risk of bias, inconsistency, indirectness of evidence, imprecision, and publication bias. The factors that can increase the certainty of the evidence are dose-response gradient, large magnitude of an effect, and effect of plausible residual confounding. Finally, we could summarize the methodology in four grades (high, moderate, low, very low).

### 2.7 Literature screening and data extraction

Two reviewers (YQ and HYJ) independently screened all literature and extracted data using EndNote X9 software. The form was utilized to gather data on the included studies, including the first author, year of publication, region where the study was conducted, number of participants in each group, sex of the patients, interventions (category of essential oils and duration of treatment), and scores on the SAS, SDS, and PSQI. Differences of opinion were mediated by a third reviewer (HBC). The identification content mainly included whether aromatherapy was inhalation therapy, whether the experimental design was RCT, and whether insomnia was comorbidity.

### 2.8 Evaluation of literature quality

Two reviewers (YQ and HYJ) categorized the risk of bias for each trial as high, low, or unclear based on the Cochrane Collaboration Risk of Bias Assessment Tool 1.0.15. The following risk of bias domains were evaluated: (1) random sequence generation (selection bias); (2) allocation concealment (selection bias); (3) blinding of participant and personnel (performance bias); (4) blinding of outcome assessment (detection bias); (5) incomplete outcome data (attrition bias), (6) selective reporting (reporting bias); and (7) any other bias. Differences of opinion were resolved by a third reviewer (HBC). All studies were assessed and categorized as high risk, low risk, or unclear risk according to their respective domains. If the number of evaluated trials exceeded five, funnel plots were employed to assess the presence of publication bias.

### 2.9 Synthesizing and examining data

Two reviewers independently used RevMan 5.3 software to synthesize and statistically analyze the efficacy data. Mean differences (MDs) with 95% confidence intervals (CIs) were presented for continuous data, and data from studies were pooled using the inverse variance method. Dichotomous data were presented as odds ratios with 95% CIs and pooled using the Mantel-Haenszel method. The heterogeneity was assessed using the Q-test and I^2^ statistic. The fixed-effects model was used in instances of low heterogeneity (I^2^<50%), while the random effects model was used in situations of moderate heterogeneity (I^2^>50%). Sensitivity analysis was used to observe the effect of a single article on heterogeneity. Subgroup analysis was performed according to the type of disease, duration of intervention, agents used, and control methods. P-values less than 0.05 were defined as statistically significant.

## 3 Results

### 3.1 Search results

**Figure 1:**
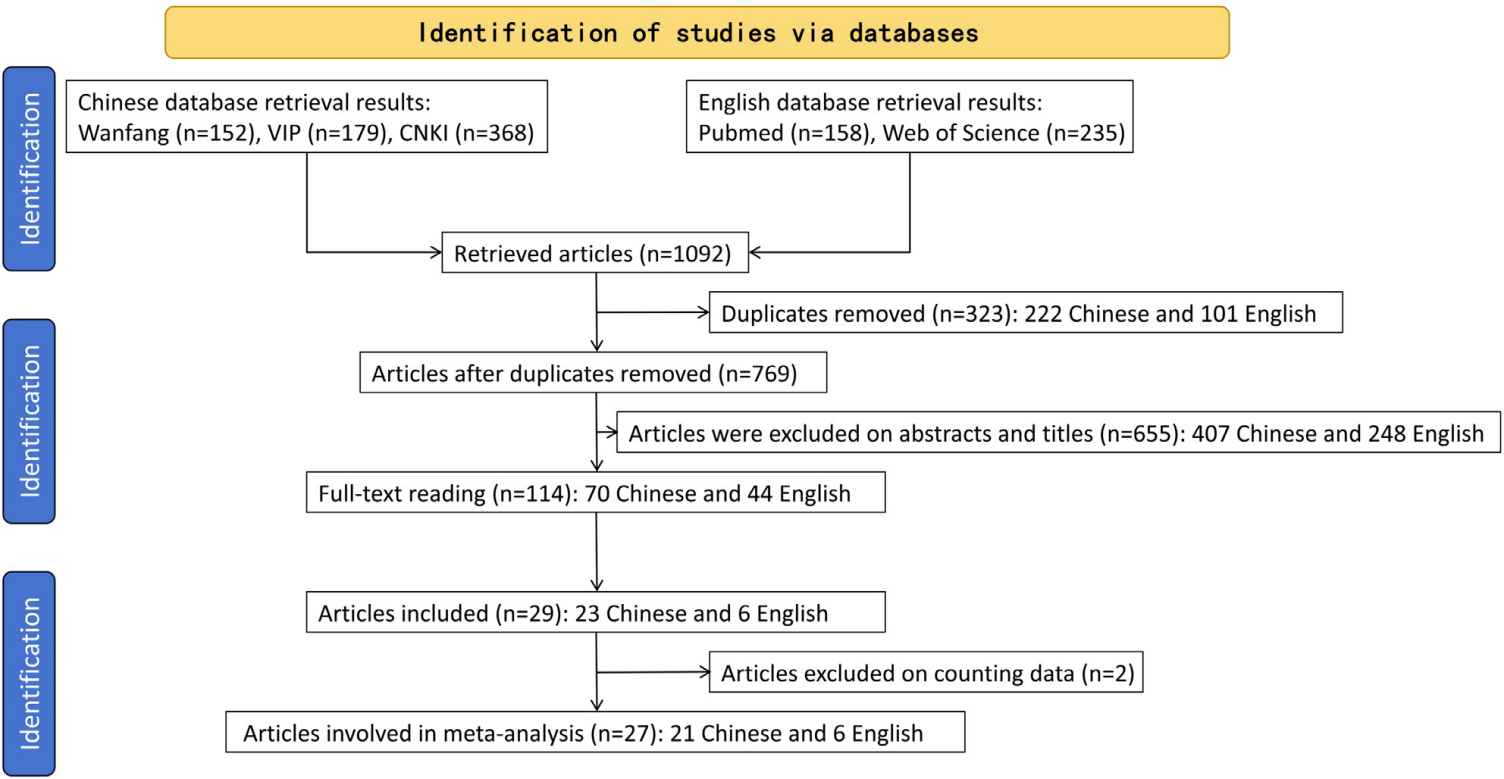
Flowchart of literature screening.

According to the retrieval methods, a total of 1092 publications that met the inclusion criteria were found, including 699 publications in Chinese and 393 publications in English. After reading the titles and abstracts, the publications that did not meet the inclusion criteria were excluded, and a total of 27 publications (26–52) met the inclusion criteria (**Figure 1**). Of these, six papers (34, 36, 39, 41, 50, 51) were master’s theses, and the other 21 papers were journal papers.

### 3.2 Basic information of the included studies

The 27 studies included a total of 2072 patients, including 1039 patients in the experimental group and 1033 patients in the control group. The basic information of the included studies is listed in **Table 1**.

**Table 1:** Basic information of the included studies.

Included studies	Diagnostic criteria for comorbid insomnia	Region	Sample size	Persons lost to follow-up	Interventions		Treatment duration (days)	Testing of purity	Testing of potency	GRADE approach
					Control group	Experimental group				
Hamzeh et al., 2020 (26)	Cancer patients	Iran	120	0	Mixing distilled water and 1% lavender essential oil for blinding	Lavender essential oil	7	low	high	low
Heydarirad et al., 2019 (27)	Cancer patients dissatisfied with their sleep quality	Iran	45	9	Routine care	Damascena rose essential oil	14	high	high	moderate
Özkaraman et al., 2018 (28)	Cancer patients receiving paclitaxel weekly	Turkey	70	NR	Routine care	Lavender essential oil	30	high	high	high
Muz et al., 2017 (29)	Hemodialysis patients with an VAS fatigue score≥3 and a PSQI score≥5.	Turkey	62	18	Routine care	1:1 mixture of lavender oil and sweet orange oil	30	high	high	low
Karadag et al., 2017 (30)	Patients with coronary artery disease and passed the first stage of the disease	Turkey	60	0	Routine care	Lavender essential oil	15	high	high	low
Hajibagheri et al., 2014 (31)	Cardiac patients	Iran	60	0	Routine care	Damascena rose essential oil	3	high	high	low
Dong et al., 2023 (32)	Patients with gastrointestinal malignancies undergoing chemotherapy	China	80	NR	Routine care	Lavender essential oil, peppermint essential oil, and bergamot essential oil were prepared as a blended essential oil at a ratio of 1:1:1.	6	middle	high	moderate
Chen et al., 2022 (33)	Breast cancer patients met the diagnostic criteria for insomnia according to both TCM and modern medicine, with a PSQI score>7, and were diagnosed with the TCM syndrome of heart-spleen deficiency	China	129	11	Routine care	Homemade lavender sachets	14	high	high	moderate
Duan et al., 2022 (34)	Patients with Alzheimer’s disease and a CDR score<3	China	51	3	Aromatherapy spray	Essential oils of lavender, sweet orange and bergamot were prepared in a 1:1:1 ratio as an essential oil compound	84	high	high	moderate
Huang et al., 2022 (35)	Colorectal cancer patients	China	120	NR	Six-character formula qigong	Lavender essential oil	28	high	high	moderate
Si et al., 2022 (36)	Patients met the diagnostic criteria for functional dyspepsia according to both TCM and modern medicine, and were diagnosed with the TCM syndrome of liver stagnation and spleen deficiency	China	126	NR	Acupoint wheat grain moxibustion	Lavender essential oil	14	high	high	moderate
Tang et al., 2022 (37)	Mild and moderate COVID-19 patients	China	84	NR	Routine care	Lavender essential oil and wild orange essential oil were prepared at a dose of 1:1 to make essential oil compound	14	high	high	moderate
Yang et al., 2022 (38)	Elderly patients with hip fracture	China	60	NR	Solid air freshener	Homemade Chinese herbal sachet	10	high	high	low
Cui et al., 2021 (39)	Maintenance hemodialysis patients met the diagnostic criteria for insomnia according to both TCM and modern medicine, and were diagnosed with the TCM syndrome of heart-kidney disharmony	China	90	0	TCM massage	Homemade Chinese herbal sachet	28	high	high	moderate
Wang et al., 2021 (40)	Chronic kidney failure uremic patients undergoing maintenance hemodialysis	China	80	NR	Routine care	Essential oils of lavender, sweet orange and bergamot were prepared in a 1:1:1 ratio as an essential oil compound.	180	high	high	moderate
Zhao et al., 2021 (41)	Maintenance hemodialysis patients	China	87	NR	Routine care	Lavender essential oil	28	high	high	moderate
Zhong et al., 2021 (42)	Elderly patients with hip fracture	China	114	NR	Routine care	Lavender essential oil	30	high	high	moderate
Zhou et al., 2021 (43)	Patients with primary liver cancer undergoing transarterial chemoembolization	China	86	NR	Routine care	Lavender essential oil	NR	high	high	moderate
Huang et al., 2020 (44)	Fracture patients undergoing open reduction and internal fixation	China	120	NR	Routine care	Homemade lavender sachets	30	high	high	moderate
Li et al., 2020 (45)	Postoperative breast cancer patients in the perichemotherapy period with a PSQI score≥8	China	100	NR	Routine care	Lavender essential oil	NR	high	high	moderate
Xie et al., 2020 (46)	Schizophrenia patients in remission period	China	64	NR	Routine care	Lavender essential oil	90	high	high	moderate
Zhang et al., 2020 (47)	Postoperative thyroid cancer patients	China	120	NR	Physiological saline placebo	Lavender essential oil	7	middle	high	low
Zhou et al., 2020 (48)	Schizophrenia patients in remission period and a PSQI score≥7	China	80	NR	Routine care	Lavender essential oil	90	low	high	moderate
Li et al., 2018 (49)	Gastric cancer patients during perioperative period	China	120	NR	Routine care	Geranium essential oil	2	high	high	moderate
Xu et al., 2018 (50)	Patients with mild to moderate acute pancreatitis undergoing non operative treatment	China	67	5	Routine care	Lavender essential oil	7	low	high	moderate
Ma et al., 2017 (51)	Post stroke depression patients with a HAMA score of 7-20 and a HAMD score of 8-35	China	59	1	Routine care	Aromatic substance extract atomization absorption	56	high	high	moderate
Qi et al., 2016 (52)	Colorectal cancer patients during perioperative period	China	69	NR	Routine care	Three essential oils of lavender, geranium and bergamot were formulated according to the ratio of 1:2:3 to make essential oil compound	10	high	high	moderate

(1) NR, not mentioned in representative literature. (2) For the purity test, the study was assessed as high if it reported purity and source, middle if it reported purity only, and low if it did not report purity. (3) For the potency test, the study was assessed as high if it reported potency and low if it did not report potency. (4) For the GRADE approach, the study was assessed as high, moderate, low, very low based on the confidence in the effectiveness estimation.

### 3.3 Evaluation of study quality

**Figure 2:**
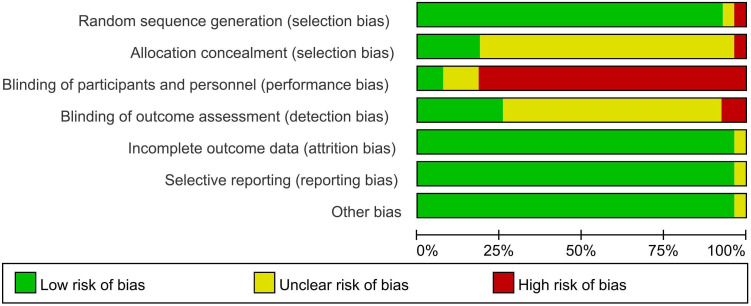
Risk of bias bar graph.

**Figure 3:**
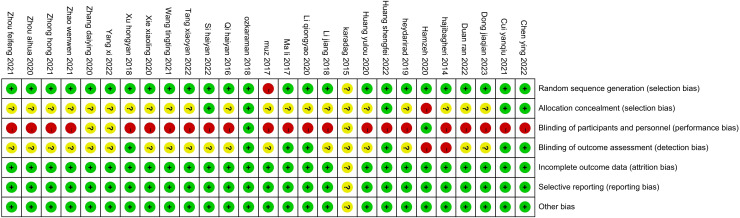
Summary of bias risks.

All 27 studies were RCTs. The studies all used correct randomization methods, including random number tables and permutation block randomization. The statistical methods of all the studies were correct, and the distribution between groups was balanced. Two studies (26, 28) reported blinding during the experiment, and six (27, 29, 33, 34, 50, 51) reported data loss, including loss to follow-up (**Figures 2**, **3**).

### 3.4 Results of the meta-analysis

#### 3.4.1 Anxiety level

**Figure 4:**
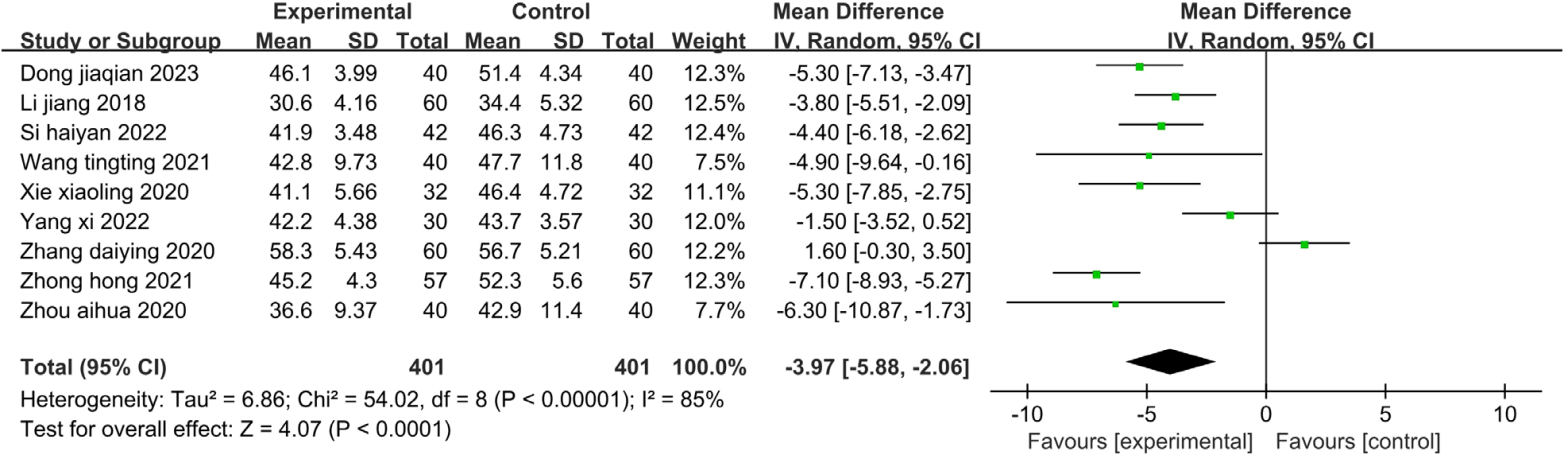
Forest plot of the meta-analysis of the effect of aromatherapy on anxiety level.

Nine studies (32, 36, 38, 40, 42, 46–49) (802 patients) reported SAS scores. There was statistical heterogeneity among the studies (P<0.00001, I^2^ = 85%). A random-effects model was used to pool the effects for the meta-analysis (**Figure 4**). The results showed that the SAS scores of the patients in the experimental group were significantly lower than those in the control group (Z=4.07, P<0.0001). Aromatherapy alleviated the anxiety of patients well. Due to the small number of included studies, subgroup analysis was not performed.

#### 3.4.2 Depression level

**Figure 5:**
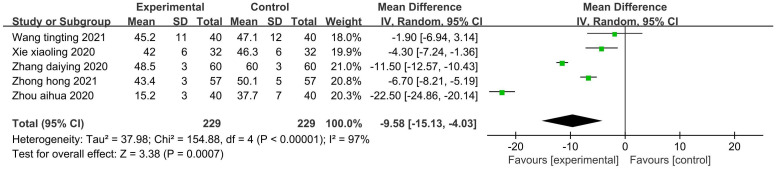
Forest plot of the meta-analysis of the effect of aromatherapy on depression level.

Five studies (40, 42, 46–48) (458 patients) reported the SDS score. There was statistical heterogeneity among the studies (P<0.00001, I^2^ = 97%). A random-effects model was used to pool the effects for the meta-analysis (**Figure 5**). The results showed that the SDS scores of the patients in the experimental group were significantly lower than those in the control group (Z=3.38, P=0.0007). Aromatherapy also did well at improving the patients’ depression status. Due to the small number of included studies, subgroup analysis was not performed.

#### 3.4.3 Sleep quality index

**Figure 6:**
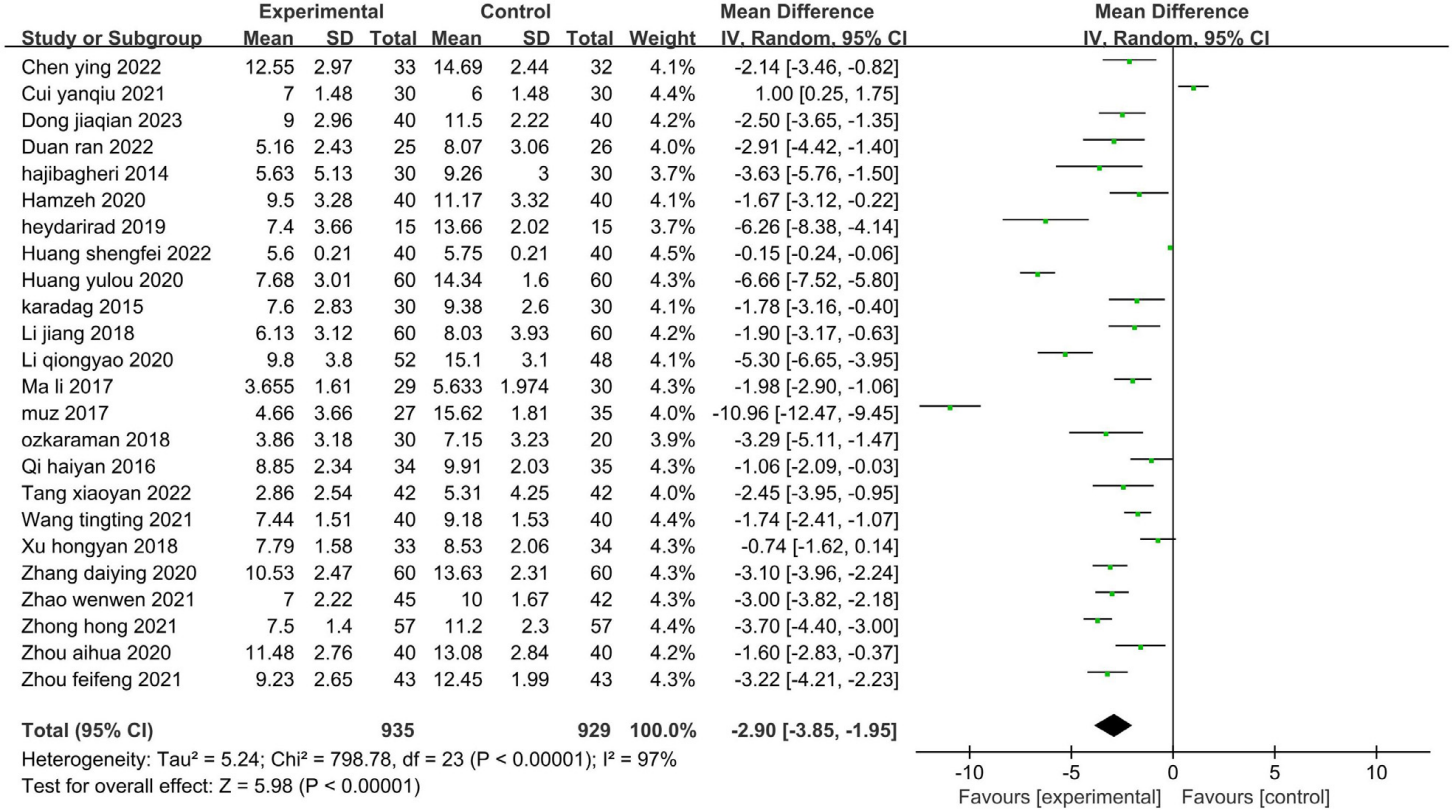
Forest plot of the meta-analysis of the effect of aromatherapy on sleep quality index.

Twenty-four studies (26–35, 37, 39–45, 47–52) (1864 patients) reported the total score on the PSQI. There was statistical heterogeneity among the studies (P<0.00001, I^2^ = 97%). A random-effects model was used to pool the effects for the meta-analysis (**Figure 6**). The PSQI scores of the patients in the experimental group were significantly lower than those in the control group (Z=5.98, P<0.00001). Thus, aromatherapy improved the patients’ sleep quality.

#### 3.4.4 Publication bias

**Figure 7:**
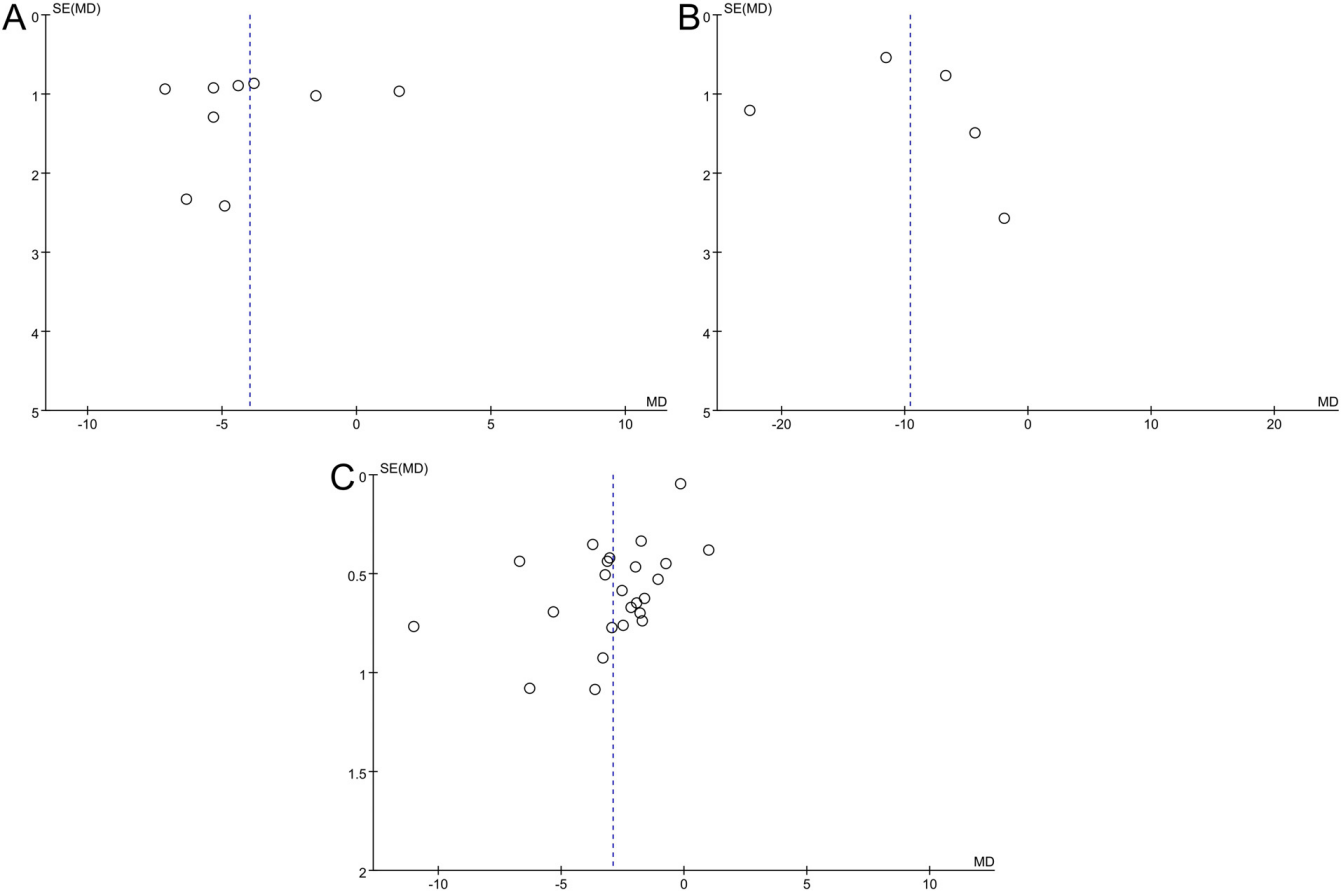
Funnel plot of the meta-analysis of the effect of aromatherapy on **(A)** anxiety level, **(B)** depression level, **(C)** sleep quality.

Visual analysis of the funnel plots revealed asymmetry, thus suggesting potential publication bias in evaluating depression-anxiety levels (**Figures 7A, B**). When exploring the studies that included the sleep quality index score, the circles in the funnel plot were scattered, and there was no obvious symmetry, suggesting that there was a possibility of publication bias (**Figure 7C**). Due to the high heterogeneity, subgroup analysis was needed to identify the factors affecting the heterogeneity.

#### 3.4.5 Subgroup analysis of sleep quality

**Figure 8:**
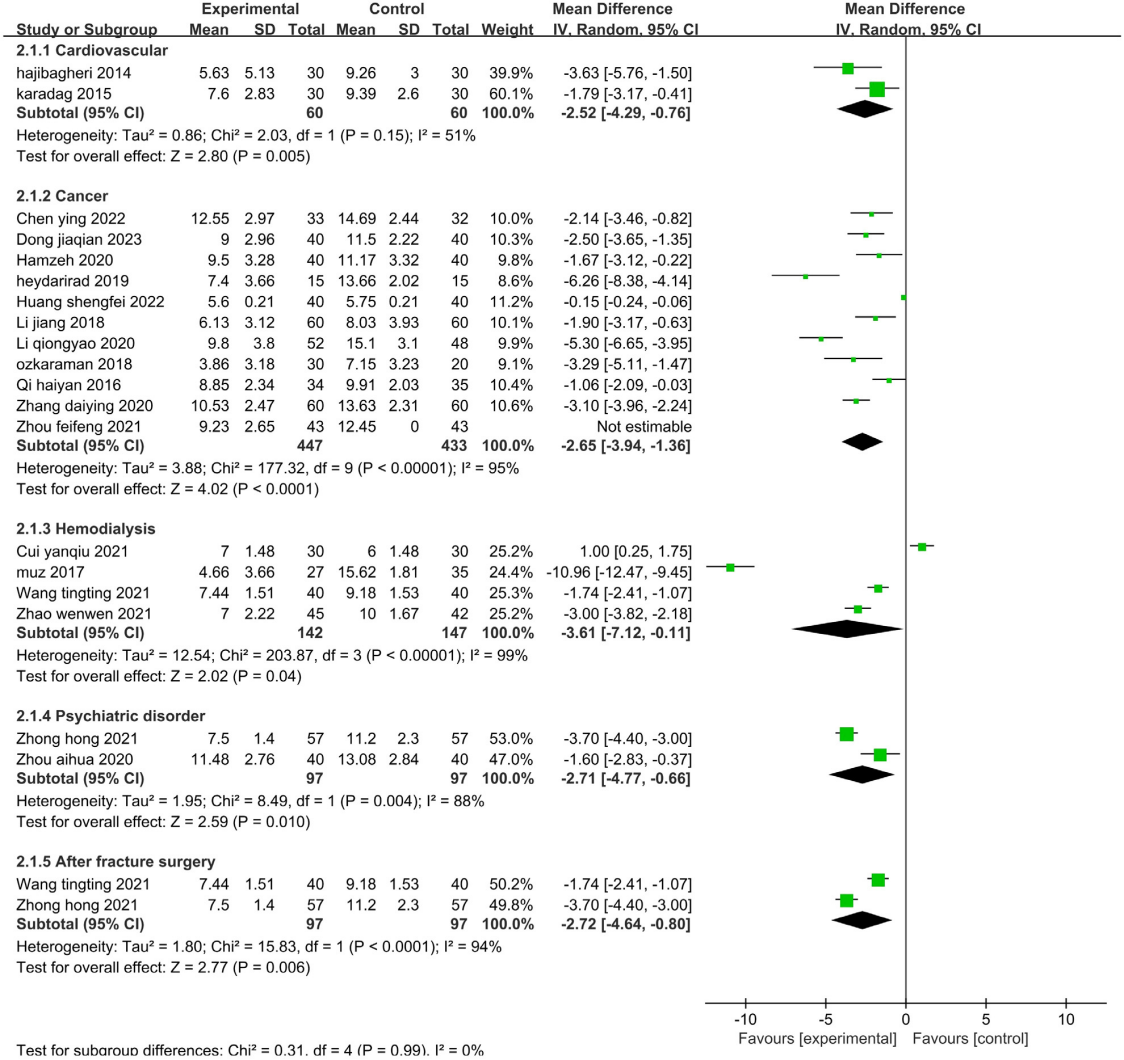
Subgroup analysis of the effect of aromatherapy on sleep quality (type of disease).

**Figure 9:**
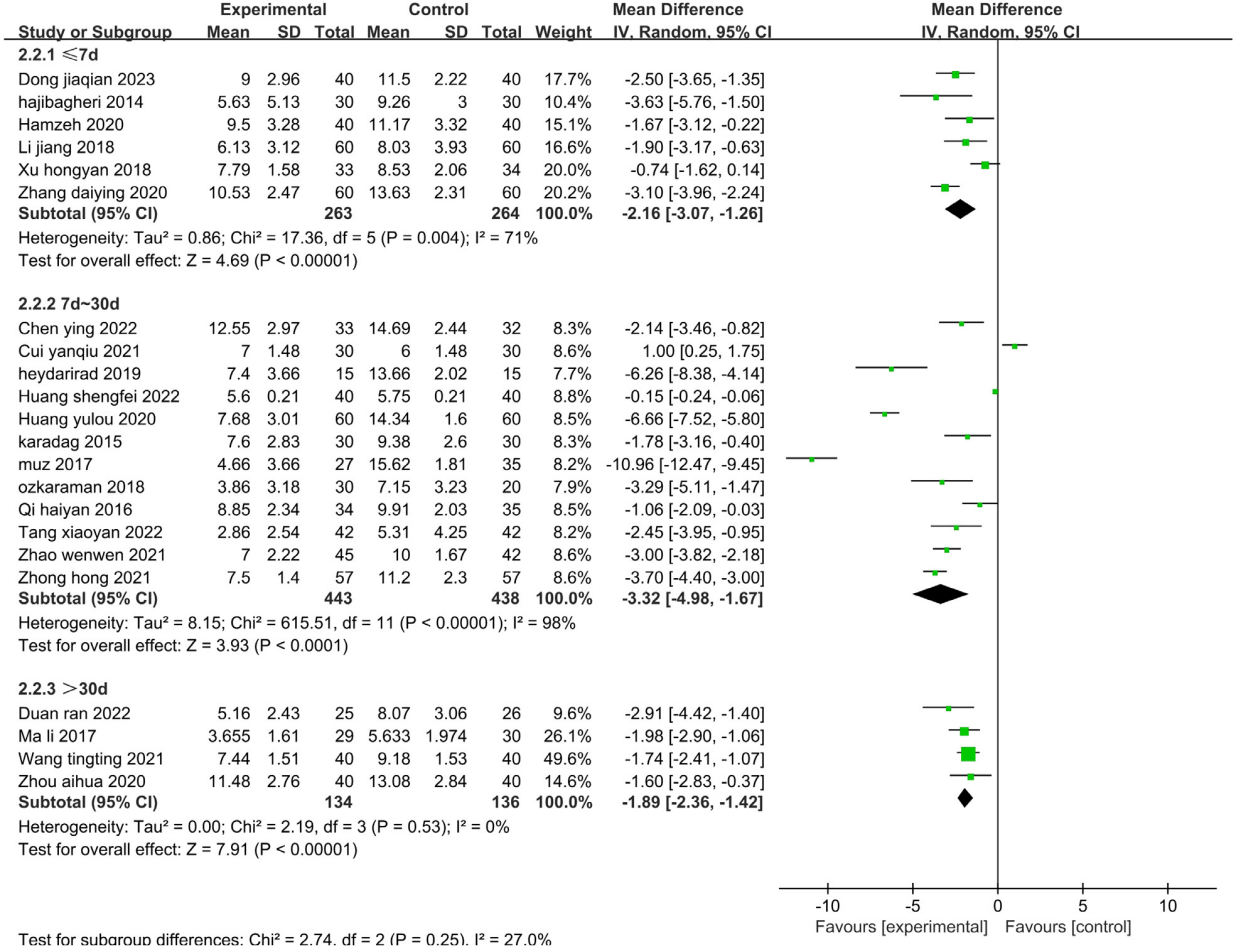
Subgroup analysis of the effect of aromatherapy on sleep quality (treatment duration).

**Figure 10:**
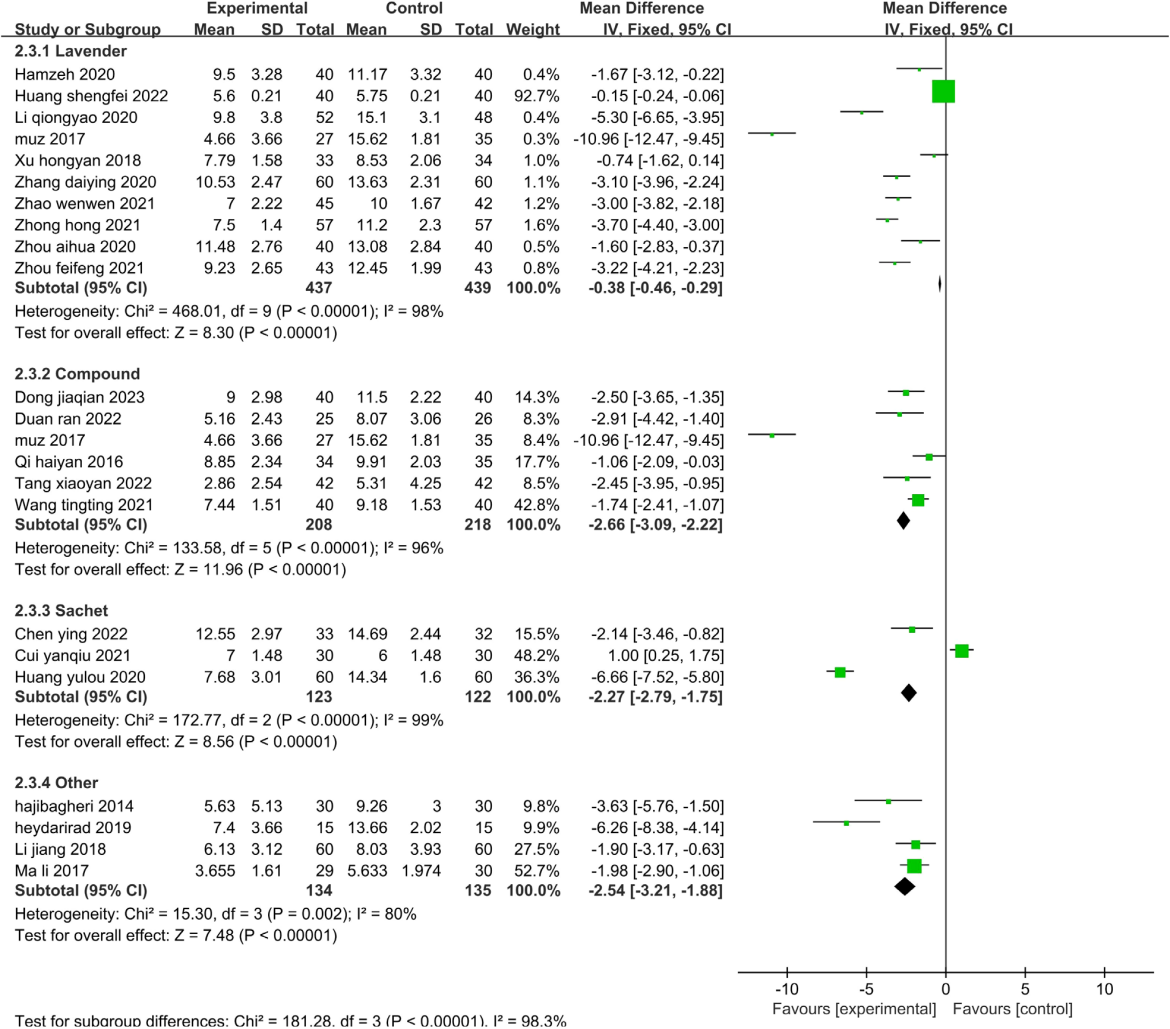
Subgroup analysis of the effect of aromatherapy on sleep quality (agent class).

**Figure 11:**
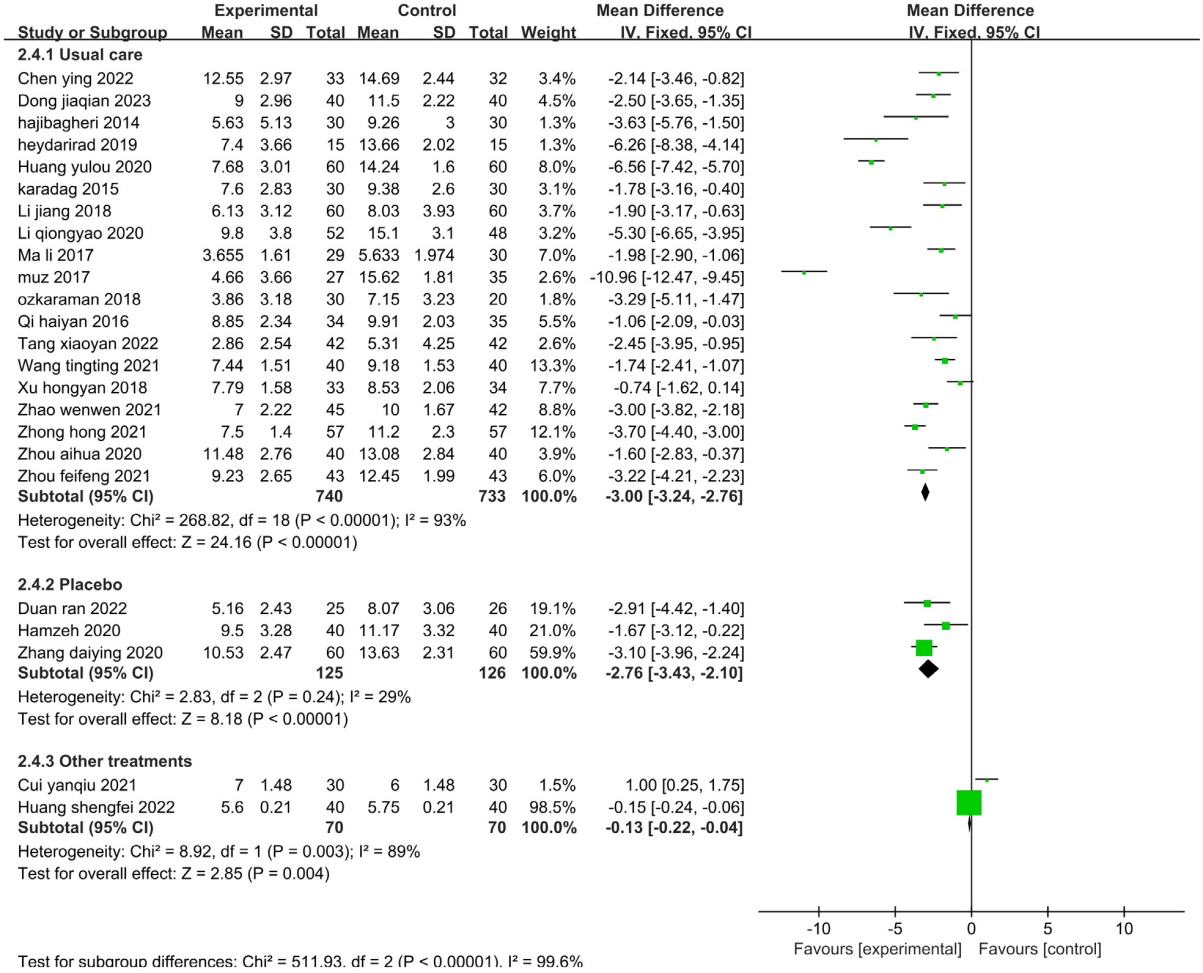
Subgroup analysis of the effect of aromatherapy on sleep quality (control method).

The sensitivity analysis showed that no single study had a great impact on heterogeneity, so the next step was to conduct subgroup analysis combined with the actual clinical work and experimental design differences. After screening the studies, we planned to conduct subgroup analysis based on the type of disease, duration of intervention, agents used, and control methods. The 19 included publications (26–33, 35, 39–43, 45, 47–49, 52) were grouped into the cardiovascular group, cancer group, hemodialysis group, psychiatric disorder group, and after fracture surgery group (**Figure 8**). The results showed that the intragroup heterogeneity was high, and the intergroup heterogeneity was low (P=0.99, I^2^ = 0%), indicating that the disease type was not an influencing factor in the heterogeneity. Twenty-two publications (26–35, 37, 39–42, 44, 47–52) were included in this analysis. The publications were grouped into three treatment durations: up to one week (≤7 d), up to one month (7–30 d), and more than one month (>30 d) (**Figure 9**). The results showed that there was high intragroup heterogeneity and low intergroup heterogeneity (P=0.25, I^2^ = 27.0%), indicating that the duration of treatment was not an influencing factor for the heterogeneity. Twenty-two publications (26, 27, 29, 31–35, 37, 39–45, 47–52) were grouped by treatment agent into the lavender group, the compound group, the sachet group, and the other group (**Figure 10**). The results showed that both the intragroup and intergroup heterogeneity was high (P<0.00001, I^2^ = 98.3%), indicating that the category of essential oils could not be excluded as not an underlying factor of the heterogeneity. Next, 23 studies (27–35, 37, 39–45, 47–52) were grouped by their control methods into the usual care group, the placebo group, or the other treatment group (**Figure 11**). The results showed that both the intragroup and intergroup heterogeneity was high (P<0.00001, I^2^ = 99.6%), so the control method could not be excluded as not an underlying factor of the heterogeneity.

#### 3.4.6 Analysis of each dimension of sleep quality

**Figure 12:**
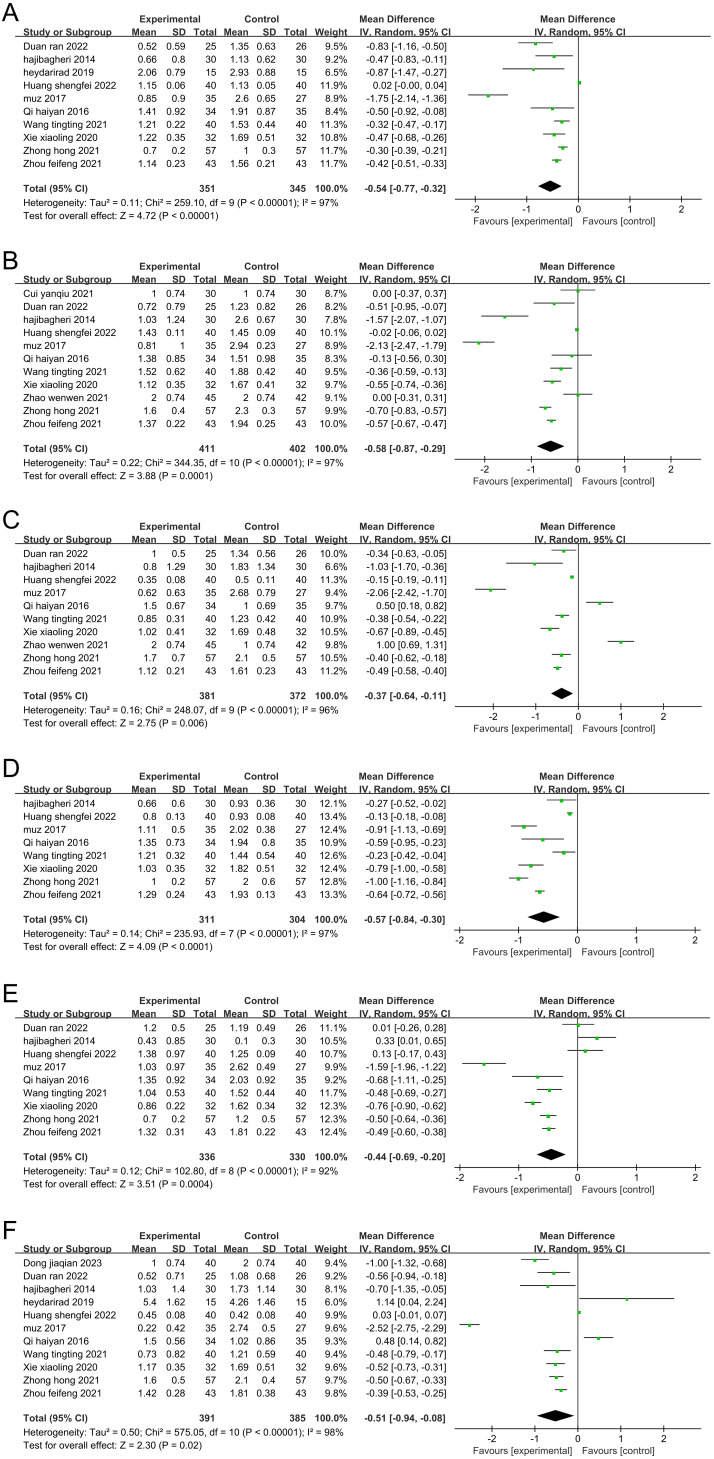
Forest plot of the meta-analysis of the effect of aromatherapy on each dimension of the sleep quality index scale **(A)** sleep quality, **(B)** time to falling asleep, **(C)** sleep efficiency, **(D)** sleep disorder, **(E)** daytime function, **(F)** sleep duration.

Meta-analysis was performed on each dimension of the sleep quality index scale. The results showed that aromatherapy had significant beneficial effects on sleep quality (**Figure 12A**), time to falling asleep (**Figure 12B**), sleep efficiency (**Figure 12C**), sleep disorder (**Figure 12D**), daytime function (**Figure 12E**), and sleep duration (**Figure 12F**) (P<0.05).

## 4 Discussion

### 4.1 Summary of evidence and analysis

Twenty-seven articles were included in this study. Our meta-analysis showed that aromatherapy well relieved anxiety and depression in the patients and improve their sleep quality during their illness. The funnel plot showed a scattered distribution of data, without obvious symmetry, suggesting that there is a certain possibility of publication bias. The pooled results were highly heterogeneous (I^2^>50%), indicating that some factors of the included studies affected the accuracy of the intragroup heterogeneity. Sensitivity analysis did not find that a single study significantly affected the heterogeneity. This indicated some stability in this result. Subgroup analysis of the sleep quality index score, which was performed in many of the included studies, confirmed that the type of disease and the duration of intervention were not factors affecting the heterogeneity, and the category of essential oils and the control method could not be excluded as not underlying factors of the heterogeneity. The results of sensitivity and subgroup analyses suggested that the heterogeneity of this study was not methodologically or statistically induced. We suspect that it may be related to the different diagnostic criteria for inclusion in the study. A meta-analysis of the six dimensions of the PSQI showed that aromatherapy can improve sleep quality (MD -0.54, 95% CI: -0.77 to -0.32, p<0.00001), shorten the time to falling asleep (MD -0.58, 95% CI: -0.87 to -0.29, p=0.0001), improve sleep efficiency (MD -0.37, 95% CI: -0.64 to -0.11, p=0.006), relieve sleep disorder (MD -0.57, 95% CI: -0.84 to -0.30, p<0.0001), enhance daytime function (MD -0.44, 95% CI: -0.69 to -0.20, p=0.0004) and lengthen sleep duration (MD -0.51, 95% CI: -0.94 to -0.08, p=0.02).

Sleep problems are common in the modern world, classified from mild to severe into sleep disorders, sleep difficulties, and insomnia (53). Primary insomnia patients showing isolated symptoms account for only 10-20% of the total number of insomnia patients. Comorbid insomnia (i.e., insomnia combined with other diseases) makes up the bulk of insomnia cases. Comorbid insomnia has many causes, which involve complex diseases, neurological function, and other factors. Therefore, modern medicine has difficulty improving this symptom based on its etiology (54). Modern studies have shown that essential oils such as lavender, rose, sweet orange, and valerian, which have sleep-promoting, sedative, and anti-anxiety effects, all contain one or both of linalool and limonene (55). Inhalation of linalool and limonene essential oils can relieve insomnia and anxiety (56). This type of treatment has long been used in traditional medicine in China, most often involving the use of vectors such as sachets and medicated pillows for disease prevention and treatment. Aromatherapy is also widely used internationally (57), showing therapeutic effects on different types of patients in pain, nausea, anxiety, depression, insomnia, and other symptoms and also benefiting patients with preoperative anxiety, tumors, palliative care, hospice care, and end of life (58–67).

### 4.2 Recommendations for further studies

Future research should aim to determine the optimal formulation of the essential oil preparation, which may include the composition and the extraction method of essential oils, the amount of essential oils to be used, and the duration of oil use. In order to further validate the long-term efficacy of inhalation aromatherapy for the treatment of comorbid insomnia, future research needs to be conducted in the following areas. First, laboratory techniques need to used to clarify the effective ingredients of aromatic traditional Chinese medicines, innovate and improve the dosage form and method of use. At the same time, large-scale clinical trials of aromatic Chinese medicines should be carried out to optimize these safe and efficient Chinese medicines. Second, syndrome analysis of traditional Chinese medicine should be conducted on secondary insomnia patients with different diseases, syndrome types and dialectical prescription should be summarized, and the clinical application potential of Chinese medicine essential oil compounds should be developed to promote the right, good, and active use of aromatherapy in first-line clinical practice. Third is to carry out more rigorous and detailed randomized controlled trial studies in China, focusing on the development of placebo oil to avoid data bias caused by breaking the blinding of patients in the control group. At the same time, patients can be guided to develop the habit of long-term use of aromatherapy, and long-term follow-up can be performed to determine its long-term efficacy.

### 4.3 Strengths and weaknesses

To the best of our knowledge, this is the first meta-analysis to investigate inhalation aromatherapy versus conventional treatment for comorbid insomnia. To reduce heterogeneity, we purposely excluded other treatment methods using aromatic essential oils. We performed subgroup analyses to identify potential sources of heterogeneity. While these analyses reduced heterogeneity between studies, our review was not without potential limitations (1): The methodological quality of the included trials was found to be low, and detailed descriptions of blinded conduct, allocation concealment, attrition, and lost cases were lacking, especially the lack of blinded design may cause a large bias in this study; (2) Most of the included trials were of short duration and did not examine the long-term efficacy of inhalation aromatherapy. Therefore, high-quality methods, further studies with large samples, and long-term interventions are still needed to evaluate the safety and effectiveness of inhalation aromatherapy in improving comorbid insomnia; (3) The level of certainty of the evidence was considered to be average; (4) The diagnostic criteria for comorbid insomnia had not been standardized at this stage. There are limitations in the studies themselves, which may have an impact on the results. Therefore, the results of this meta-analysis should be treated with caution.

## 5 Conclusion

In summary, this article provides a systematic evaluation of the effectiveness of inhalation aromatherapy in treating comorbid insomnia. Studies have shown that aromatherapy has good clinical efficacy in treating comorbid insomnia, improving the quality of life of patients during the disease period. Moreover, the safety of clinical application of aromatherapy is relatively high, and no adverse reactions have been reported in the selected literature. Due to the low quality evaluation of the included studies, this conclusion still needs to be approached with caution. In addition, there is a lack of blinding and independent testing of the purity and efficacy of aromatic essential oils in the design of research methods, which still needs large scale clinical research and high quality evidence in the future to confirm this conclusion. We call on more medical workers to invest in this research direction and truly address the urgent needs of patients.

## Data Availability

The original contributions presented in the study are included in the article/supplementary material. Further inquiries can be directed to the corresponding authors.
